# *MSTO1* mutations cause mtDNA depletion, manifesting as muscular dystrophy with cerebellar involvement

**DOI:** 10.1007/s00401-019-02059-z

**Published:** 2019-08-29

**Authors:** S. Donkervoort, R. Sabouny, P. Yun, L. Gauquelin, K. R. Chao, Y. Hu, I. Al Khatib, A. Töpf, P. Mohassel, B. B. Cummings, R. Kaur, D. Saade, S. A. Moore, L. B. Waddell, M. A. Farrar, J. K. Goodrich, P. Uapinyoying, S.H. S. Chan, A. Javed, M. E. Leach, P. Karachunski, J. Dalton, L. Medne, A. Harper, C. Thompson, I. Thiffault, S. Specht, R. E. Lamont, C. Saunders, H. Racher, F. P. Bernier, D. Mowat, N. Witting, J. Vissing, R. Hanson, K. A. Coffman, M. Hainlen, J. S. Parboosingh, A. Carnevale, G. Yoon, R. E. Schnur, Care4Rare Canada Consortium, K. M. Boycott, J. K. Mah, V. Straub, A. Reghan Foley, A. M. Innes, C. G. Bönnemann, T. E. Shutt

**Affiliations:** 1 Neuromuscular and Neurogenetic Disorders of Childhood Section, National Institute of Neurological Disorders and Stroke, National Institutes of Health, Bethesda, MD USA; 2 Department of Biochemistry and Molecular Biology, University of Calgary, Calgary, Canada; 3 Division of Clinical and Metabolic Genetics, Department of Paediatrics, The Hospital for Sick Children, University of Toronto, Toronto, ON Canada; 4 Division of Neurology, Department of Paediatrics, The Hospital for Sick Children, University of Toronto, Toronto, ON Canada; 5 Center for Mendelian Genomics, Program in Medical and Population Genetics, Broad Institute of MIT and Harvard, Boston, MA USA; 6 John Walton Muscular Dystrophy Research Centre, Institute of Genetic Medicine, Newcastle University, Newcastle upon Tyne, UK; 7 Department of Pathology Carver College of Medicine, The University of Iowa, Iowa City, IA USA; 8 Kids Neuroscience Centre, Kids Research, The Children’s Hospital at Westmead, Sydney, NSW 2145 Australia; 9 Discipline of Child and Adolescent Health, Faculty of Medicine and Health, The University of Sydney, Westmead, NSW 2145 Australia; 10 Department of Neurology, Sydney Children’s Hospital, Sydney, NSW Australia; 11 UNSW Sydney, School of Women’s and Children’s Health, Sydney, NSW Australia; 12 Department of Paediatrics and Adolescent Medicine, Queen Mary Hospital, The University of Hong Kong, Hong Kong SAR, China; 13 School of Biomedical Science, The University of Hong Kong, Hong Kong SAR, China; 14 Oregon Health and Science University, Neuromuscular Program, Doernbecher Children’s Hospital, Portland, OR USA; 15 Department of Neurology, University of Minnesota, Minneapolis, MN USA; 16 Division of Human Genetics, The Children’s Hospital of Philadelphia, Philadelphia, USA; 17 Department of Neurology, Virginia Commonwealth University, Children’s Hospital of Richmond at VCU, Richmond, VA USA; 18 Department of Pediatrics, University of California San Diego, San Diego, CA USA; 19 Department of Pathology and Laboratory Medicine, Children’s Mercy Hospital, Kansas City, USA; 20 Center for Pediatric Genomic Medicine, Children’s Mercy Hospital, Kansas City, USA; 21 University of Missouri-Kansas City School of Medicine, Kansas City, USA; 22 Department of Medical Genetics and Alberta Children’s Hospital Research Institute, Cumming School of Medicine, University of Calgary, Calgary, AB Canada; 23 Department of Medical Genetics, Sydney Children’s Hospital, Sydney, NSW Australia; 24 Department of Neurology, University Hospital Rigshospitalet, Copenhagen, Denmark; 25 Department of Pediatrics, Children’s Mercy Hospital, Kansas City, USA; 26 Division of Neurology, Children’s Mercy Hospital, Kansas City, USA; 27 GeneDx, Gaithersburg, MD USA; 28 Children’s Hospital of Eastern Ontario Research Institute, University of Ottawa, Ottawa, Canada; 29 Care4Rare Research Consortium, Ottawa, Canada; 30 Departments of Pediatrics, Section of Neurology, University of Calgary, Calgary, AB Canada; 31 Newcastle Hospitals NHS Foundation Trust, Newcastle upon Tyne, UK; 32 Department of Medical Genetics, Alberta Children’s Hospital Research Institute, Hotchkiss Brain Institute, University of Calgary, Calgary, Canada; 33 Research for Genetic Medicine, Children’s National Medical Center, Washington, DC USA

**Keywords:** MSTO1, Mitochondrial fusion, Cerebellar atrophy, Muscular dystrophy, MtDNA depletion

## Abstract

*MSTO1* encodes a cytosolic mitochondrial fusion protein, misato homolog 1 or MSTO1. While the full genotype–phenotype spectrum remains to be explored, pathogenic variants in *MSTO1* have recently been reported in a small number of patients presenting with a phenotype of cerebellar ataxia, congenital muscle involvement with histologic findings ranging from myopathic to dystrophic and pigmentary retinopathy. The proposed underlying pathogenic mechanism of *MSTO1*-related disease is suggestive of impaired mitochondrial fusion secondary to a loss of function of MSTO1. Disorders of mitochondrial fusion and fission have been shown to also lead to mitochondrial DNA (mtDNA) depletion, linking them to the mtDNA depletion syndromes, a clinically and genetically diverse class of mitochondrial diseases characterized by a reduction of cellular mtDNA content. However, the consequences of pathogenic variants in *MSTO1* on mtDNA maintenance remain poorly understood. We present extensive phenotypic and genetic data from 12 independent families, including 15 new patients harbouring a broad array of bi-allelic *MSTO1* pathogenic variants, and we provide functional characterization from seven *MSTO1*-related disease patient fibroblasts. Bi-allelic loss-of-function variants in *MSTO1* manifest clinically with a remarkably consistent phenotype of childhood-onset muscular dystrophy, corticospinal tract dysfunction and early-onset non-progressive cerebellar atrophy. MSTO1 protein was not detectable in the cultured fibroblasts of all seven patients evaluated, suggesting that pathogenic variants result in a loss of protein expression and/or affect protein stability. Consistent with impaired mitochondrial fusion, mitochondrial networks in fibroblasts were found to be fragmented. Furthermore, all fibroblasts were found to have depletion of mtDNA ranging from 30 to 70% along with alterations to mtDNA nucleoids. Our data corroborate the role of MSTO1 as a mitochondrial fusion protein and highlight a previously unrecognized link to mtDNA regulation. As impaired mitochondrial fusion is a recognized cause of mtDNA depletion syndromes, this novel link to mtDNA depletion in patient fibroblasts suggests that MSTO1-deficiency should also be considered a mtDNA depletion syndrome. Thus, we provide mechanistic insight into the disease pathogenesis associated with *MSTO1* mutations and further define the clinical spectrum and the natural history of *MSTO1*-related disease.

**Electronic supplementary material:**

The online version of this article (10.1007/s00401-019-02059-z) contains supplementary material, which is available to authorized users.

## Introduction

Mitochondria maintain and express their own genome (mtDNA), typically present in 100–1000 copies per cell and organized into nucleoprotein structures known as nucleoids [5, 18]. The mtDNA encodes thirteen subunits of the oxidative phosphorylation (OXPHOS) machinery that are essential for mitochondrial respiration and ATP production [39]. The relative amount of mtDNA per cell varies in a tissue-specific manner, as an adequate number of mtDNA copies must be maintained to support aerobic respiration and meet cellular energetic demands [24]. As such, reduction in the total amount of mtDNA clinically manifests as severe multi-systemic abnormalities to which energy-demanding organs, such as the brain and muscles, are particularly susceptible.

MtDNA depletion syndromes are a clinically and genetically diverse class of mitochondrial diseases characterized by a reduction of mitochondrial genomes [14, 33]. Of the 15 formally defined mtDNA depletion syndromes listed in The Online Mendelian Inheritance in Man database (OMIM) [4], most are caused by pathogenic variants in proteins that are required for mtDNA replication (POLG, C10orf2, MGME1, and TFAM) or those that are necessary to maintain mitochondrial deoxyribonucleoside triphosphates (dNTP) pools (TK2, DGUOK, RRM2B, TYMP, SUCLA2, SUCLG1, AGK, MPV17 and SLC25A) [14, 48]. However, pathogenic variants in proteins that regulate the processes of mitochondrial fusion and fission have also been linked to mtDNA depletion. These include the fusion protein OPA1 (OMIM 616896), as well as MFN2 and DNM1L, which are essential for fusion and fission, respectively [25, 34, 41, 46, 49].

While defects in mtDNA replication or impaired maintenance of mitochondrial dNTP pools are expected to lead to mtDNA depletion, it is less clear how mitochondrial fusion and fission are involved in the regulation of mtDNA. Nonetheless, abnormalities in mtDNA integrity and nucleoid distribution have been demonstrated in several models of defective fusion. Mitochondrial membrane fusion is orchestrated by the activity of large GTPases, including Mitofusin 1 and 2 (MFN1, MFN2) localized to the outer membrane, and Optic Atrophy 1 (OPA1) in the inner mitochondrial membrane. In yeast, cells lacking the Mitofusin homolog Fzo1p suffer from complete loss of mtDNA [21]. Meanwhile, knockout fibroblasts for OPA1, MFN1 and/or MFN2 display fragmented mitochondrial networks with altered nucleoid distribution whereby some fragments are devoid of mitochondrial genomes [10]. Finally, in humans, pathogenic variants in *MFN2* and *OPA1* cause severe mtDNA depletion and multiple mtDNA deletions [41, 46, 49]. Patients with pathogenic variants in *MFN2* or *OPA1* that are associated with mtDNA impairments have been reported to present with a phenotype of early-onset ataxia, hypotonia, axonal sensorimotor neuropathy, optic atrophy and hearing loss [2, 3, 22, 41, 46, 49].

Recently, MSTO1 was described as a cytosolic mitochondrial fusion protein, and pathogenic variants in *MSTO1* have been reported to cause ataxia, muscle weakness, cerebellar atrophy and pigmentary retinopathy [16, 31, 36]. Notably, these phenotypic features have all been previously reported in mtDNA depletion syndromes [3, 14, 22]. To date, five independent studies have described *MSTO1* variants in 12 patients from seven families [6, 16, 31, 36]. While pathogenic *MSTO1* variants have been linked to impairments in mitochondrial fusion, the consequence of these variants on mtDNA maintenance and its associated clinical spectrum has not been studied extensively.

In this study, we present an extensive phenotypic characterization of 15 new patients from 12 families harbouring a broad array of bi-allelic pathogenic variants in *MSTO1* confirming a remarkably consistent and ultimately recognizable clinical phenotype. Additionally, in cultured fibroblasts from seven patients with *MSTO1*-related disease, we demonstrate loss of MSTO1 protein, significantly fragmented mitochondrial networks, enlarged lysosomal vacuoles, depletion of mtDNA, and alterations to mtDNA nucleoids. Therefore, we demonstrate that bi-allelic loss-of-function variants in the mitochondrial fusion protein MSTO1 impair mtDNA maintenance and fusion and result in mtDNA depletion in fibroblasts, which we establish is associated with a remarkably consistent clinical spectrum of MSTO1-deficiency.

## Materials and methods

### Human subjects and samples

Patients were ascertained through their local neurology and genetics clinics. F2 was identified through GeneMatcher [45]. Written informed consent and age-appropriate assent for study procedures and photographs were obtained by a qualified investigator (protocol 12-N-0095 approved by the National Institute of Neurological Disorders and Stroke, National Institutes of Health, Research Ethics Board of the Hospital for Sick Children, REB # 1000009004: SCHN Human Ethics Committee 10/CHW/45, University of Calgary Conjoint Health Research Ethics Board). Medical history was obtained and clinical evaluations, including brain MRI and muscle biopsy, were performed as part of the standard diagnostic evaluation. Muscle biopsy slides and available electron microscopy images (EM) were reviewed by investigators. DNA, muscle and skin biopsy samples were obtained according to standard procedures.

### Cell culture

Control and patient fibroblasts were generated from skin biopsies and cultured in MEM media (Gibco, 11095080) containing l-Glutamine and supplemented with 10% foetal bovine serum (FBS). HeLa cells were grown in DMEM (Gibco, 11965092) supplemented with 10% FBS. Cells were maintained at 37 °C and 5% CO_2_. HeLa cells were transfected with a mammalian expression vector containing MSTO1-V5 (DNASU, HsCD00440595) using Lipofectamine 3000 (Thermo Fisher Scientific, L3000015) according to manufacturer’s instructions. Briefly, cells were seeded at 4.5 × 10^5^ in 6-well plates and allowed to grow overnight. The following day, 2 µg of plasmid were used for transfections and cells were left to grow for 24 h before harvesting.

For genetic rescue experiments, fibroblasts were electroporated with an MSTO1-P2A-mCherry construct using the Amaxa Nucleofector II system (Lonza). The *MSTO1* sequence was cloned into an AmCyan-P2A-mCherry construct (AmCyan-P2A-mCherry was a gift from Ilpo Huhtaniemi, Addgene plasmid # 45350; http://n2t.net/addgene:45350; RRID:Addgene_45350) [40], replacing the mCyan sequence with the *MSTO1* sequence. An empty vector containing only mCherry was also generated. Fibroblasts grown to 70–80% confluence were resuspended in OptiMEM media. Next, 1 × 10^6^ cells in 100 µL media and 2 µg of plasmid DNA were transferred to a sterile 2 mm electroporation cuvette (VWR 89047-208) and electroporated using the A-024 program. Cells were then plated onto 35 mm glass bottom dishes or in 100 mm dishes and incubated for 48 h prior to further analysis.

### Cell sorting

Following electroporation with either mCherry empty vector or MSTO1-P2A-mCherry, approximately 4 × 10^6^ fibroblast cells were sorted for red fluorescence (and MSTO1 expression) using a 130-µm nozzle on a BD FACSAria Fusion (FACSAriaIII) cytometer (BD Biosciences), supported by FACSDiva Version 8.0.1. Genomic DNA was subsequently purified from control and patient mCherry-positive cells as described below.

### Western blot

For Western analyses, 3 × 10^5^ cells were seeded in 100 mm plates, allowed to grow for 2–3 days, harvested by trypsin digestion, washed with 1X phosphate-buffered saline (PBS) and lysed with RIPA buffer containing protease inhibitors. Total cell lysates (50 µg) were resolved on SDS-PAGE gels and transferred onto PVDF membranes. Blots were subsequently probed with the following antibodies (1:1000 dilution unless otherwise indicated): anti-MSTO1 (Genetex, GTX105110) (1:500), anti-V5 (Millipore, AB3792), anti-Mitofusin1 (Cell Signalling, 14739), anti-Mitofusin2 (Abnova, H00009927-M03), anti-Opa1 (BD Bioscience, 612606), anti-Actin (Sigma A5316), anti-HSP60 (Cell Signalling, 12165), VDAC1 (Abcam, ab14734) and the appropriate horseradish peroxidase (HRP)-conjugated secondary antibodies (1:3000). Blots were incubated with Clarity ECL substrate (Biorad, 1705061) and imaged on an Amersham Imager AI600.

### mtDNA copy number analysis

Total genomic DNA (gDNA) (nuclear and mitochondrial DNA) was extracted from control and patient fibroblasts using the PureLink Genomic DNA Mini Kit (Thermo Fisher Scientific, K182001) according to manufacturer’s instructions. Relative mtDNA copy number was analysed by real-time quantitative PCR (qPCR) using the QuantStudio 6 Flex Real-Time PCR system (Thermo Fisher Scientific). Primer sequences specific to mtDNA, nuclear-encoded housekeeping gene 18S and thermocycling conditions were performed as described previously [13].

QPCR reactions were prepared to a total of 20 uL per reaction containing 10 uL PowerUp SYBR Green Master Mix (Thermo Fisher Scientific, A25742), 100 ng gDNA and 500 nM forward and 500 nM reverse primers. MtDNA copy number relative to 18S was analysed using the delta delta Ct method and represented as percent control [32]. Relative mtDNA copy number was presented as mean ± SD from at least three independent biological replicates and unpaired, 2-tailed Student’s *t* tests were used to determine statistical significance.

### Immunofluorescence staining

Fibroblasts were seeded on 12 mm glass coverslips (no. 1.5) at 2X10^4^ cells and allowed to grow for 24 h. Cells were subsequently fixed and stained with primary antibodies against TOMM20 (Santa Cruz Biotechnology, FL-145) and LAMP1 (Santa Cruz Biotechnology, 18821) in addition to appropriate Alexafluor-conjugated secondary antibodies (Thermo Fisher Scientific) at 1:1000 as done previously [42].

### Live cell imaging

In order to visualize mitochondrial DNA nucleoids, fibroblasts were stained with PicoGreen (Thermo Fisher Scientific, P7581) as described previously [7]. Briefly, cells were seeded on glass bottom dishes (Mattek, P35G-1.5-14-C) at 8 × 10^4^ and incubated overnight. Approximately 1 h prior to imaging, cells were stained with PicoGreen at 3 µL/mL for 30–45 min at 37 °C. MitoTracker Red dye (50 nM) (Thermo Fisher Scientific, M7512) or MitoTracker Deep Red dye (50 nM) (ThermoFischer Scientific, M22426) was also added to the media to visualize mitochondrial networks. The media containing dyes was aspirated, cells were washed four times in pre-warmed 1XPBS, and fresh pre-warmed media was added to the cells.

### Microscopy

Images from both fixed and live samples were acquired on an Olympus spinning disc confocal system (Olympus SD OSR) (UAPON 100XOTIRF/1.49 oil objective) operated by Metamorph software. A cell Vivo incubation module was used to maintain cells at 37 °C and 5% CO_2_ during live cell imaging.

### Image analysis

#### Mitochondrial networks

Mitochondrial morphology was qualitatively analysed by classifying networks into one of four categories, as previously described [16]. For each fibroblast line, at least 50 cells were scored, and the analyses were performed on three independent replicates. Results represent mean ± SD, and *P* values were based on unpaired, 2-tailed Student’s *t* tests.

#### MtDNA nucleoids

Mitochondrial DNA nucleoid size and number were analysed in 35 fibroblast cells from each patient line and control using the particle analysis tool in ImageJ FIJI [43]. First, images were acquired using the same acquisition parameters, scaled and binarized. For each cell, a region of interest (ROI) encompassing the entire mitochondrial network was selected. Then, in binarized mtDNA nucleoid images, the particle analysis tool was used to measure surface area and total nucleoid counts within the selected ROI. Nuclear signal was excluded from the analysis. Nucleoid sizes are presented as the average size of all nucleoids per cell in a violin plot, highlighting the distribution of quantified mtDNA nucleoid sizes. The non-parametric Kolmogorov–Smirnov (K–S) test was used to determine statistical significance regarding the distribution of nucleoid sizes. The relative frequencies of nucleoid sizes were assessed in each quantified cell and the percentage of large nucleoids (> 0.2 µm^2^) in control and patient fibroblasts was plotted. Nucleoid counts represent mean ± SD, and *P* values were based on unpaired, 2-tailed Student’s *t* tests.

#### Lysosomes

The presence of enlarged lysosomal aggregates was quantified from confocal images of patient and control cells by scoring the number of cells with normal vs. enlarged lysosomes. At least 50 cells were analysed per fibroblast line from two independent replicates. Results represent mean ± SD, and *P* values were obtained from unpaired, 2-tailed Student’s *t* tests.

## Results

### Clinical characteristics

The clinical presentation of the 15 patients, which includes nine females and six males, is summarized in Table 1, with ages ranging from 6 to 52 years at the time of most recent examination. There were two families with more than one affected relative: Family 1 (F1) consists of three affected sisters [P1, P2, P3; p.(Asp236His); p.(Arg279His)], and Family 2 (F2) consists of two affected siblings [P4, P5; p.(Gly420ValfsX2); p.(Arg256Gln)] (Fig. 2d). Family history was non-contributory in the remaining patients of the cohort.

**Table 1:** Clinical characteristics

	F1P1	F1P2	F1P3	F2P4	F2P5	P6	P7	P8	P9	P10	P11	P12	P13	P14	P15
Mutations	c.706G>C; p.D236H c.836G>A; p.R279H	c. 706G>C; p.D236H c.836G>A; p.R279H	c.706G>C; p.D236H c. 836G>A; p.R279H	c.1259delG; p.G420VfsX2; c.767G>A; p.R256Q	c.1259delG; p.G420VfsX2; c.767G>A; p.R256Q	c.706G>C; p.D236H c.651C>G; p.F217L	c.1350G>C; p.L450F; Deletion	c.706G>C; p.D236H c.651C>G; p.F217L	c.1433A>G; p.Y478C; Missing second allele	c.707A > G; p.D236G c.836G>A; p.R279H	c.706G>C; p.D236H c.836G>A; p.R279H	c.1033C>T; p.R345H c.971C>T; p.T324l	c.651C>G; p.F217L c.651C>G; p.F217L	c.651C>G; p.F217L c.835C>T; p.R279C	c.40G>A; p.G14R c.225_ 230del; p.L76_S77del
Sex/age (years)	F/19	F/17	F/12	M/23	F/21	M/37	M/9	M/6	F/19	F/16	F/20	F/6	M/23	M/52	F/7
Ethnicity	Caucasian	Caucasian	Caucasian	African	African	Caucasian	Caucasian	Caucasian	Chinese	Caucasian/Hispanic	Caucasian	Chinese	Caucasian	Caucasian	Native American
Symptom onset	6 months	6 months	6 months	1 year	Congenital	Congenital	Congenital	15 months	1 year	Congenital	2 years	1 year	18 months	< 7 years	3 years
Motor development	Delayed; walked between 2.5 and 3 years	Delayed; walked at 2 years	Delayed; walked at 2 years	Delayed; walked at 6 years	Delayed; walked at 8 years	Delayed; walked at 22 months	Delayed; pulled-to- stand at 18 months	Delayed; walked at 23 months	Delayed; walked at 4 years	Normal	Normal	Delayed; walked at 2.5 years	Normal	Unknown	Normal
Muscle strength (MRC)	Proximal weakness (3/5 range)	Proximal weakness; mild facial weakness	Proximal weakness (hips and shoulders 3-3 +/5)	UE proximal weakness (3/5 range)	UE proximal weakness (3/5 range)	Proximal weakness (3/5 range); mild facial weakness	Proximal weakness (3/5 range); mild facial weakness	Mild proximal weakness	Proximal weakness	Proximal weakness (3/5 range); mild facial weakness	Proximal weakness	Proximal weakness	Proximal weakness (3/5 range)	Mild proximal weakness	Proximal weakness
Corticospinal tract involvement	Clonus	Reflexes 3 + UE, 4 + LE with spreading	Clonus; reflexes 3 + with spreading	Increased tone in UE and LE; reflexes 3+	Increased tone in LE; reflexes 3+	Normal (reflexes 2+)	Babinski, clonus, increased tone in LE; reflexes 2 + (reduced triceps)	Normal (reflexes 2+)	Normal (reflexes 2+)	Babinski, clonus, spastic catch; reflexes 3+	Clonus; reflexes 3+ with spreading	Normal (reflexes 2+)	Absent deep tendon reflexes	Unknown	Normal (reflexes 2+)
Gait	Wide-based, waddling-like, difficulties with tandem gait	Slightly ataxic, mildly Trendelenburg, difficulties with tandem gait	Wide-based, waddling-like, difficulties with tandem gait	Ataxic, wide-based; cane for long distances	Ataxic, wide-based with assistance	Waddling-like	Wide-based, waddling-like with bilateral foot drop and circumduction; unable to do tandem gait	Wide-based; unable to do tandem gait	Ataxic, wide-based; unable to do tandem gait	Wide-based, waddling-like	Trendelenburg, difficulties with tandem gait	Ataxic, wide-based, unsteady	Wheelchair for longer distances	Ambulatory	Ataxic, wide-based
Cerebellar symptoms	+	+ Mild dysmetria	+ Mild dysmetria	+ Dysmetria; tremor	+ Dysmetria; tremor	–	+ Dysmetria	+ Mild dysmetria	+ Mild dysmetria	+ Slight tremor	–	+ Dysmetria and tremor	–	**–**	+ Dysmetria
Cognitive involvement	**–**	Learning difficulties	Learning difficulties	Learning difficulties	Learning difficulties	**–**	–	**–**	Learning difficulties	**–**	–	Learning difficulties	Learning difficulties	Learning difficulties	Learning difficulties
Speech involvement	Speech delay	Speech delay	Dysarthria	Dysarthria	Dysarthria	–	Speech delay	Dysarthria	Speech delay	Dysarthria	–	Dysarthria	**–**	Dysarthria	Dysarthria; Speech delay
Swallowing difficulties	**–**	**–**	**–**	+	+	**–**	+	–	+	+	–	–	–	Unknown	**–**
CK (U/L)	500–788 (16 years)	959 (2 years)	334 (2 years)	4550 (3 years), 3487 (18 years)	1494 (16 months), 3196 (17 years)	1192 (37 years)	4387 (5 years)	600-951	4,029 (16 years)	1451 (9 years), 1629 (12 years)	1200-1300 (3 years)	1867 (18 months), 2249 (3 years)	1450 (14 years)	1450 (47 years)	107 (7 years)
EMG	Not performed	Not performed	Not performed	Myotonia (15 years)	Myotonia (14 years)	Not performed	Not performed	Complex repetitive discharges (3 years)	Not performed	Myotonia (9 years and 13 years)	Not performed	Not performed	Not performed	Not performed	Not performed
Muscle biopsy	R vastus lateralis (7 years): marked variation in fiber size with evidence of fiber type grouping	Not performed	Not performed	R quadriceps (2 years): severe atrophy of type I fibres and fiber splitting	Not performed	(2.5 years): Marked variation in fiber size, occasional internalized nuclei and atrophy of type I fibres	R vastus lateralis (20 months): marked variation in fiber size, increased internalized nuclei, rare degenerating and regenerating fibres, significant whorled fibres; EM: subtle Z-line streaming	(4 years): Marked variation in fiber size, increased internalized nuclei, type I fiber predominance, rare regenerating fibres	L deltoid (16 years): marked variation in fiber size, increased internalized nuclei; EM: subtle Z-line streaming and increased subsarcolemal mitochondria with normal morphology	L biceps (9 years): marked variation in fibre size, fiber splitting, significant whorled fibres and evidence of moth-eaten appearance; EM: increased subsarcolemal mitochondria with abnormal morphology	L quadriceps (3 years): marked variation in fibre size, significant whorled fibre, increased internalized nuclei, occasional degenerating and regenerating fibres, type I fibre predominance	R quadriceps (3 years): marked variation in fiber size, degenerating and regenerating fibers, increased internalized nuclei	(13 years): marked variation in fiber size	(39 years): marked variation in fiber size	Not performed
Brain MRI	(6 years): Cerebellar atrophy involving vermis and both hemispheres	(5 years): Cerebellar atrophy involving vermis and both hemispheres	(2 years): Cerebellar atrophy involving vermis and both hemispheres	(2 years): Cerebellar atrophy involving vermis and both hemispheres; (15 years): moderate to marked diffuse vermis and cerebellar volume loss; mild increased FLAIR and T2 signal of the cerebellar white matter surrounding the fourth ventricle	(16 months): Cerebellar atrophy involving vermis and both hemispheres; (14 years): no progression of cerebellar atrophy; increased T2 signal in the peritrigonal white matter	Not performed	(9 years): Cerebellar atrophy involving vermis and both hemispheres	(1 year): Cerebellar atrophy involving vermis and both hemispheres; (5 years): no progression of cerebellar atrophy	(16 years): Cerebellar atrophy involving vermis and both hemispheres	(9 years): Mild cerebellar atrophy and pontine hypoplasia; (16 years): no progression of cerebellar atrophy	(20 years): Cerebellar atrophy involving vermis and both hemispheres	(1 year): Cerebellar atrophy involving both hemispheres; (6 years): slight progression of cerebellar atrophy	Not performed	Not performed	(5 years): Cerebellar atrophy involving vermis and both hemispheres; (6 years): no progression of cerebellar atrophy
Muscle MRI	Not performed	Not performed	Not performed	Not performed	Not performed	(37 years): Diffuse fatty infiltration of all upper leg muscles except for the semimembranosus and biceps femoris; relative sparing of the soleus and the flexor hallucis longus with prominent atrophy of the lateral gastrocnemius bilaterally	(9 years): Fatty infiltration of all muscles bilaterally with relatively increased involvement of the adductor magnus bilaterally	(6 years): Fatty infiltration of all muscles with relatively increased involvement of the sartorius when compared with the gracilis and prominent atrophy of the lateral gastrocnemius bilaterally	Not performed	(16 years): Fatty infiltration of all muscles with increased involvement of the rectus femoris and relatively increased involvement of the sartorius when compared with the gracilis and prominent atrophy of the lateral gastrocnemius bilaterally	Not performed	Not performed	Not performed	Not performed	Not performed
FVC % predicted	60%	62%	69%	Not performed	Not performed	77% (37 years)	68% (7 years)	64% (6 years)	Not performed	71% (16 years)	64% (18 years)	Not performed	79% (14 years)	62% (39 years)	Not performed
Echocardiogram	NL (5 years)	Not performed	Not performed	NL	NL	NL (37 years)	NL (9 years)	NL (6 years)	NL (21 years)	NL (16 years)	NL	NL (3 years)	NL (14 years)	NL (52 years)	NL (4 years)
Other	Short stature	Short stature	Short stature	None	None	None	None	None	Longstanding esotropia and short stature	(16 years): Anisoastigmatism with mild ambylopia of the right eye	None	Short stature and microcephaly	None	Vasovagal syncope with asystole	None

Patients typically presented with hypotonia and delayed motor milestones with first symptoms recognized between birth and three years of age. All but five patients presented with delayed motor milestones and then subsequently achieved independent ambulation with gaits characterized as waddling-like and wide-based across the entire cohort. All 15 patients reported either relatively slow progression or no progression of their muscle weakness. At the time of the last examination, all were found to have predominantly proximal weakness. Cerebellar symptoms manifested as dysmetria in 11, gait ataxia in nine and abnormal speech in 12, which included a history of speech delay in five and dysarthria in eight patients, respectively. Corticospinal tract manifestations including increased tone, the presence of a spastic catch, clonus and/or increased deep tendon reflexes were observed in eight patients. A history of learning difficulties was reported in nine patients while none of the patients were found to have major cognitive involvement. None of the patients had a history of seizures, cataracts, hearing or cardiac involvement.

**Fig. 1:**
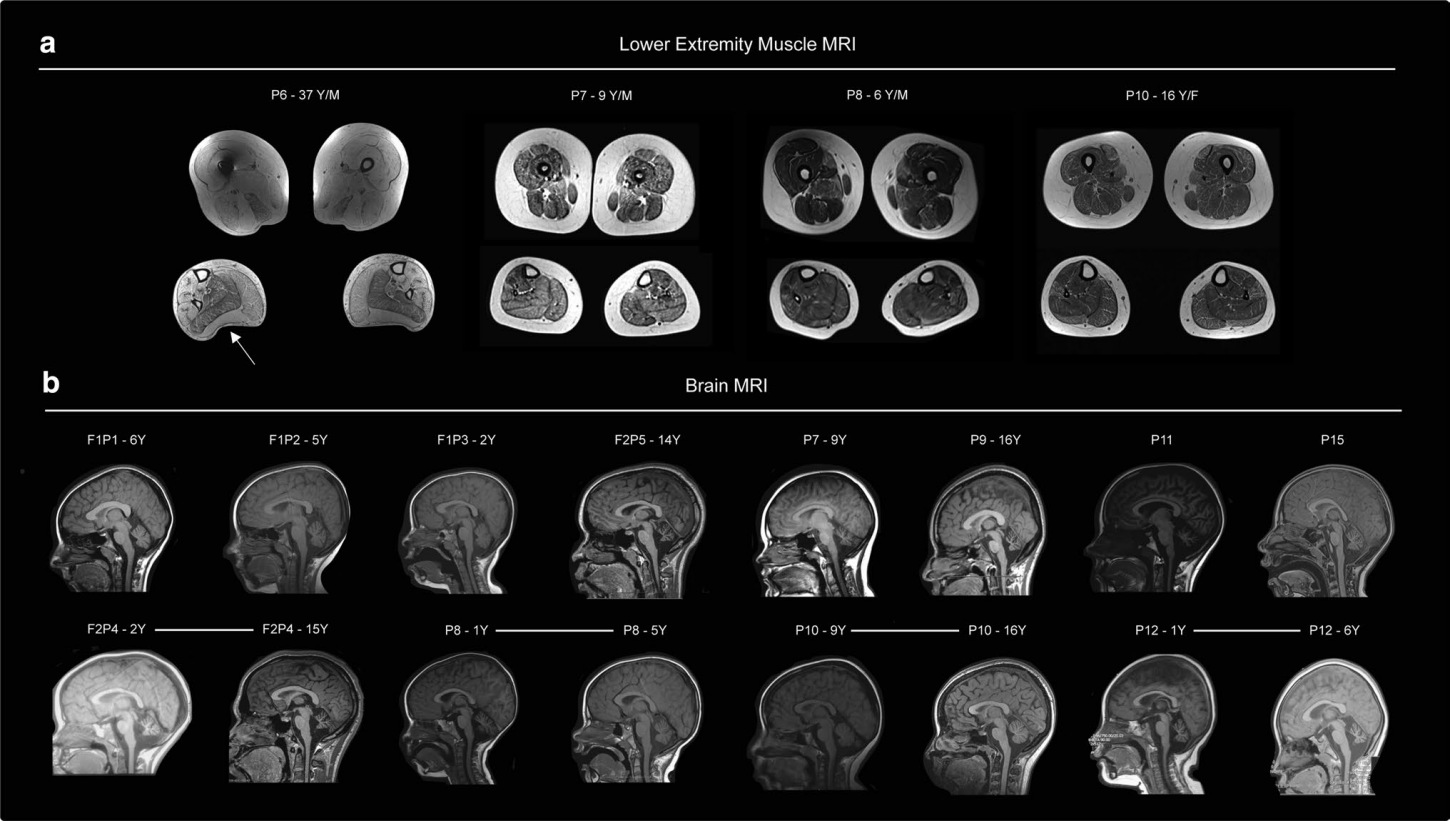
Muscle and brain imaging. **a** Lower extremity muscle MRI of patients P6 [p.(Asp236His); p.(Phe217Leu)], P7 [p.(Leu450Phe); deletion], P8 [p.(Phe217Leu); p.(Asp236His)] and P10 [p.(Asp236Gly); p.(Arg279His)] at ages 37 years, 9 years, 6 years and 16 years, respectively. Abnormal signal intensity of muscles such as the posterior gastrocnemius muscle in patient P6 (white arrow), reflects muscle breakdown and replacement with adipose tissue. **b** Brain MRI completed in 12 patients consistently demonstrates moderate-to-severe cerebellar volume loss or hypoplasia involving the vermis and both hemispheres. Repeat MRI images were available in patients P4 [p.(Gly420ValfsX2); p.(Arg256Gln)], P8 [p.(Phe217Leu); p.(Asp236His)], P10 [p.(Asp236Gly); p.(Arg279His)] and P12 [p.(Arg345His); p.(Thr324Ile)] demonstrate mild [P12] to no progression [P4, P8 and P10] of cerebellar volume loss over time (second row)

Electrophysiological studies were available for four patients. Three [P4: p.(Gly420ValfsX2); p.(Arg256Gln), P5: p.(Gly420ValfsX2); p.(Arg256Gln), P10; p.(Asp236Gly); p.(Arg279His)] of the four had evidence of myotonia on EMG, and complex repetitive discharges were present in P8 [p.(Phe217Leu); p.(Asp236His)]. Lower extremity muscle MRI imaging was available for four patients and revealed a spectrum of involvement ranging from severe involvement of the muscles of the upper leg with apparent fatty replacement of all muscles except for the semimembranosus and the biceps femoris (P6; p.(Asp236His); p.(Phe217Leu)) to mild fatty infiltration of upper and lower leg muscles [P10; p.(Asp236Gly); p.(Arg279His)] (Table 1; Fig. 1a). Pulmonary function testing was performed in nine patients and revealed reduced forced vital capacity (FVC) measurements, ranging from 60 to 79% predicted. Serum creatine kinase (CK) levels were significantly increased in all patients, except for P15, ranging from 300 to 5000 U/L. Echocardiogram was normal in the 11 patients who underwent this screening evaluation.

Overall, we recorded a remarkably consistent clinical phenotype of primary motor developmental delay, fairly stable mostly proximal muscle weakness caused by a muscular dystrophy, mild cerebellar findings of dysmetria, ataxia and dysarthria based on a stable congenital cerebellar atrophy, mild pyramidal signs and evidence for some degree of speech delay and learning disability in some. Meanwhile, major cognitive involvement, seizures, retinopathy, optic atrophy or hearing loss were not seen.

### Neuroimaging characteristics

Brain MR imaging was available for 12 patients and consistently showed moderate to severe cerebellar atrophy/hypoplasia involving the vermis and both hemispheres in all 12 (Fig. 1b). Four of the brain MRIs had been performed in patients before age 2 years, which revealed significant decrease in cerebellar volume. A lack of progression of cerebellar volume loss was confirmed through repeat imaging available in four patients. Patient 12 [p.(Arg345His); p.(Thr324Ile)] showed mild progression of cerebellar volume loss between ages one and 6 years.

### Muscle histopathology and electron microscopy

**Fig. 2:**
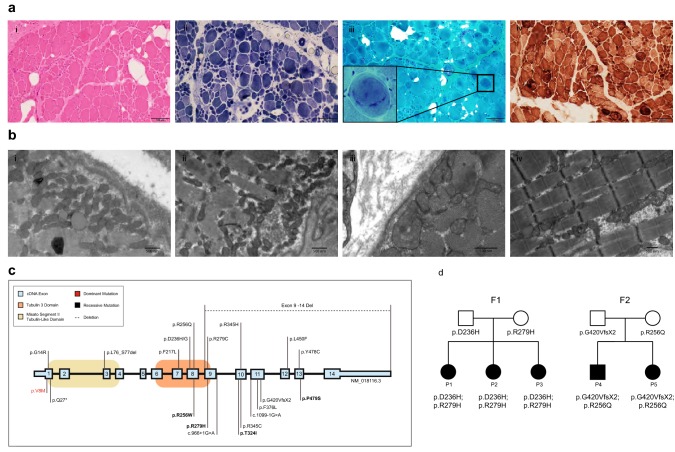
Muscle biopsy, *MSTO1* pathogenic variants and pedigrees. **a** Histology findings from the vastus lateralis muscle biopsy of P7 [p.(Leu450Phe); deletion] at age 20 months include internalized nuclei on hematoxylin and eosin (H&E) staining (white arrow) (i) and variation in fiber size on nicotinamide dinucleotide (NADH) staining (ii) and whorled fibres evident on Gömöri trichrome (inset) (iii) and COX staining (white arrow) (iv). **b** Muscle biopsy electron microscopy (EM) findings are notable for aggregates of subsarcolemmal mitochondria in both P9 [p.(Tyr478Cys); missing)] (i and ii) and P10 [p.(Asp236Gly); p.(Arg279His)] (iii and iv) and non-specific mitochondrial morphologic abnormalities (variations in mitochondrial shape and size) in P10. **c** Schematic of new and reported human *MSTO1* pathogenic variants. Shown in numbered light blue squares are cDNA exons (RefSeq isoform NM_018116.3 of *MSTO1*). Corresponding known protein domains are shown in orange (tubulin 3 domain) and beige (Misato segment II tubulin-like domain). Variants written in black text are recessive; the single mutation in red has been previously reported to cause dominantly inherited *MSTO1*-related disease. The top half of the figure depicts novel variants reported in this publication; the bottom half of the figure depicts variants which have been previously reported. Bolded variants depict previously reported mutations that were also present in our cohort. The dotted line depicts a large deletion (exons 9-14). **d** Pedigree of two families consistent with recessive inheritance of *MSTO1* pathogenic variants

Muscle biopsies were performed in ten patients and were consistent with a dystrophic process with evidence of variation in fibre type size, a mild degree of necrosis and regeneration, internalized nuclei and whorled fibres (Fig. 2a). Electron microscopy analysis was performed in three patients. Aggregates of subsarcolemmal mitochondria were noted in patients P9 [p.(Tyr478Cys); missing] and P10 [p.(Asp236Gly); p.(Arg279His)]. There was also evidence of non-specific mitochondrial morphologic abnormalities (variations in mitochondrial shape and size) seen in patients P9 and P10 (Fig. 2b).

### Molecular results

Using whole exome sequencing (WES), we identified apparent homozygous or compound heterozygous variants in *MSTO1* (NM_018116.3) in 15 patients from 12 independent families consistent with the bi-allelic recessive mode of inheritance (Fig. 2c). Parental DNA for segregation testing was not available for P13, who was found to be apparently homozygous for the common p.(Phe217Leu) variant. Six of these missense variants have not yet been reported. The p.(Gly420ValfsX2) frameshift variant was recently reported [31]. There were three apparent recurring mutation hotspots (p.Asp236, p.Arg279, p.Phe217), which were identified as five, four and four independent alleles, respectively. The p.(Arg279His) variant had been previously reported in heterozygosity with a second pathogenic allele in three families [23, 31].

Trio WES identified an apparently homozygous p.(Leu450Phe) *MSTO1* pathogenic variant in P7. Targeted sequencing confirmed that this variant was paternally inherited, while the mother was found to be negative for the variant. Subsequent testing using exon-level oligo CGH array identified a presumed maternally inherited deletion in P7 encompassing at least exons 9-14 of the *MSTO1* gene and extending to include both the *YY1AP1* as well as the *DAP3* genes (Genomic Coordinates: arr[GRCh37] 1q22(155582110_155708204)x1). Recessive pathogenic variants in *YY1AP1* have been reported in association with Grange syndrome (OMIM 607860). *DAP3* has not yet been associated with human disease.

Trio WES for P9 identified a heterozygous p.(Tyr478Cys) *MSTO1* variant inherited from a clinically unaffected father. Whole genome sequencing (WGS) in P9 confirmed this heterozygous missense variant but did not identify a second *MSTO1* allele in compound heterozygosity. Exon-level oligo CGH testing for P9 was normal. RNA sequencing (RNA-seq) analysis to identify any possible transcriptional aberrations in *MSTO1* for P9 was inconclusive, and no splice aberrations were identified in P9. Attempts to evaluate allele balance at the hg19: chr1:155583319 variant via RNA-seq were unsuccessful due to insufficient coverage in the region, likely due to the presence of a highly homologous pseudogene.

Parental segregation testing for all *MSTO1* variants was consistent with bi-allelic recessive inheritance, except for P9 (p.(Tyr478Cys); missing) in whom the presumed maternally inherited allele has not yet been identified, and for P13 (p.(Phe217Leu); p.(Phe217Leu)) in whom parental DNA was not available. Variants identified were predicted to be damaging and either absent or extremely rare (allele frequency below 0.00005) in Genome Aggregation Database (GnomAD) and Exome Aggregation Consortium (ExAC) except for the p.(Arg279His) variant. This particular variant was listed with an allele frequency of 0.00019 in ExAC and 0.00026 in GnomAD with one reported homozygous individual [29]. *MSTO1* variants are scattered throughout the gene and do not seem to cluster in a specific MSTO1 domain (Fig. 2c).

### Characterization of patient fibroblasts

**Fig. 3:**
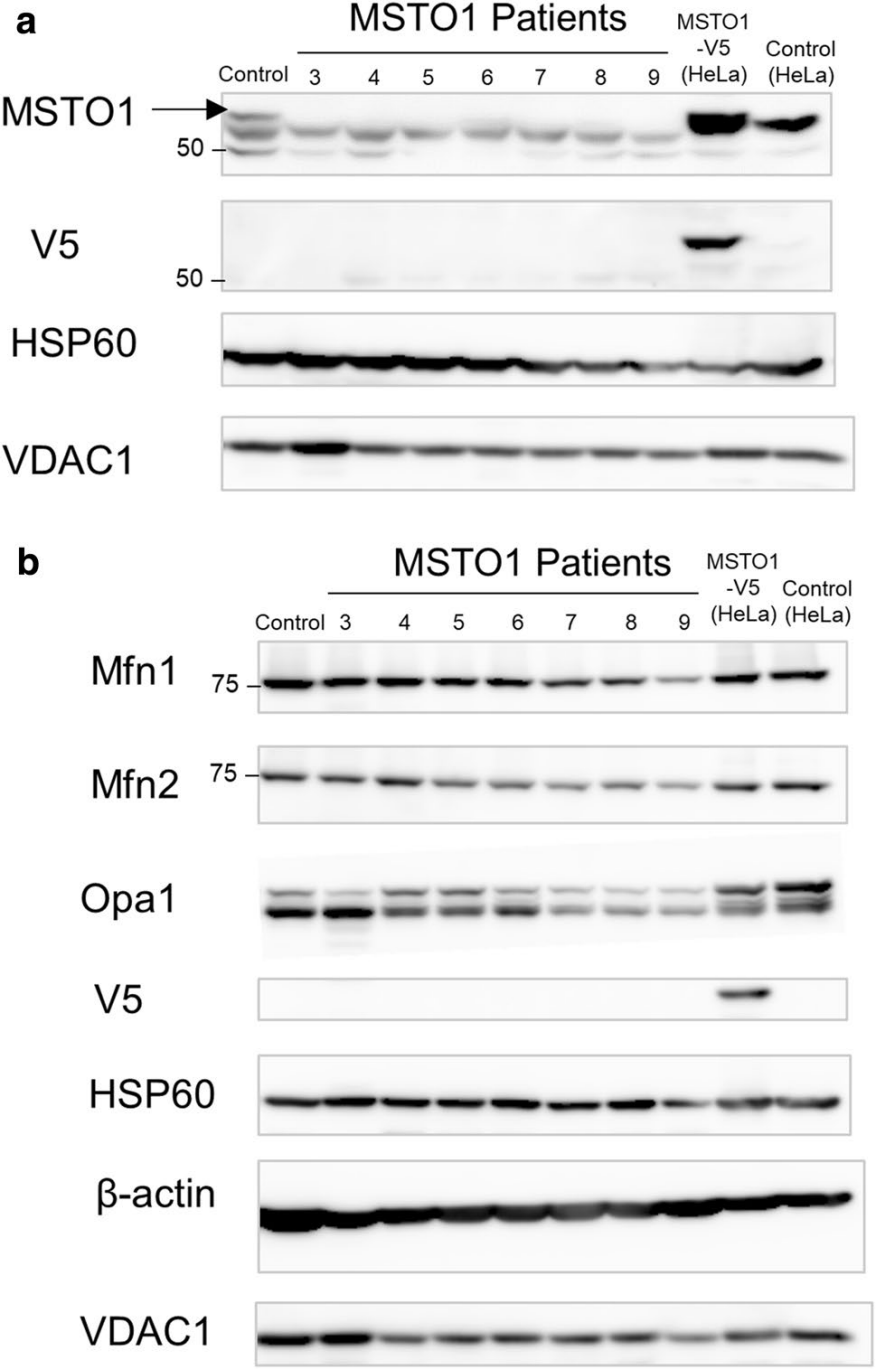
Pathogenic variants lead to MSTO1 protein instability. **a** Western blot analysis of total cell lysates from control and patient fibroblast. As a control, total cell lysates from HeLa cells overexpressing MSTO1-V5 or empty vector were also included. Blots were probed with antibodies against endogenous MSTO1, VDAC1, HSP60 and V5. Black arrow corresponds to endogenous MSTO1 protein further verified in HeLa cell lysates; meanwhile, bands underneath are nonspecific. **b** Western blot analysis of cell lysates as in **a**. Blots were probed against fusion proteins (Mfn1, Mfn2 and Opa1) and loading controls

In order to further investigate the effect of *MSTO1* mutations, we examined the expression of MSTO1 protein by immunoblotting. As the MSTO1 antibody we used detects multiple bands in fibroblast cells, we confirmed the size of the correct band, which migrates at the predicted size of 62 kDa, corresponding to MSTO1 protein in HeLa cells overexpressing a V5-epitope-tagged-MSTO1 or an empty vector (Fig. 3a). Our data show that the MSTO1 protein is undetectable in all patient fibroblasts, (*n* = 7) suggesting that pathogenic variants affect protein expression and/or stability (Fig. 3a). Although this observation was expected in cells with large deletions encompassing the *MSTO1* gene [e.g. P7; p.(Leu450Phe); deletion)], it is intriguing that all patient fibroblasts containing combinations of missense mutations exhibit a similar cellular phenotype. It is also worth noting that *MSTO1* variants do not have gross effects on the expression of mitochondrial fusion proteins (MFN1/2 and OPA1) (Fig. 3b).

**Fig. 4:**
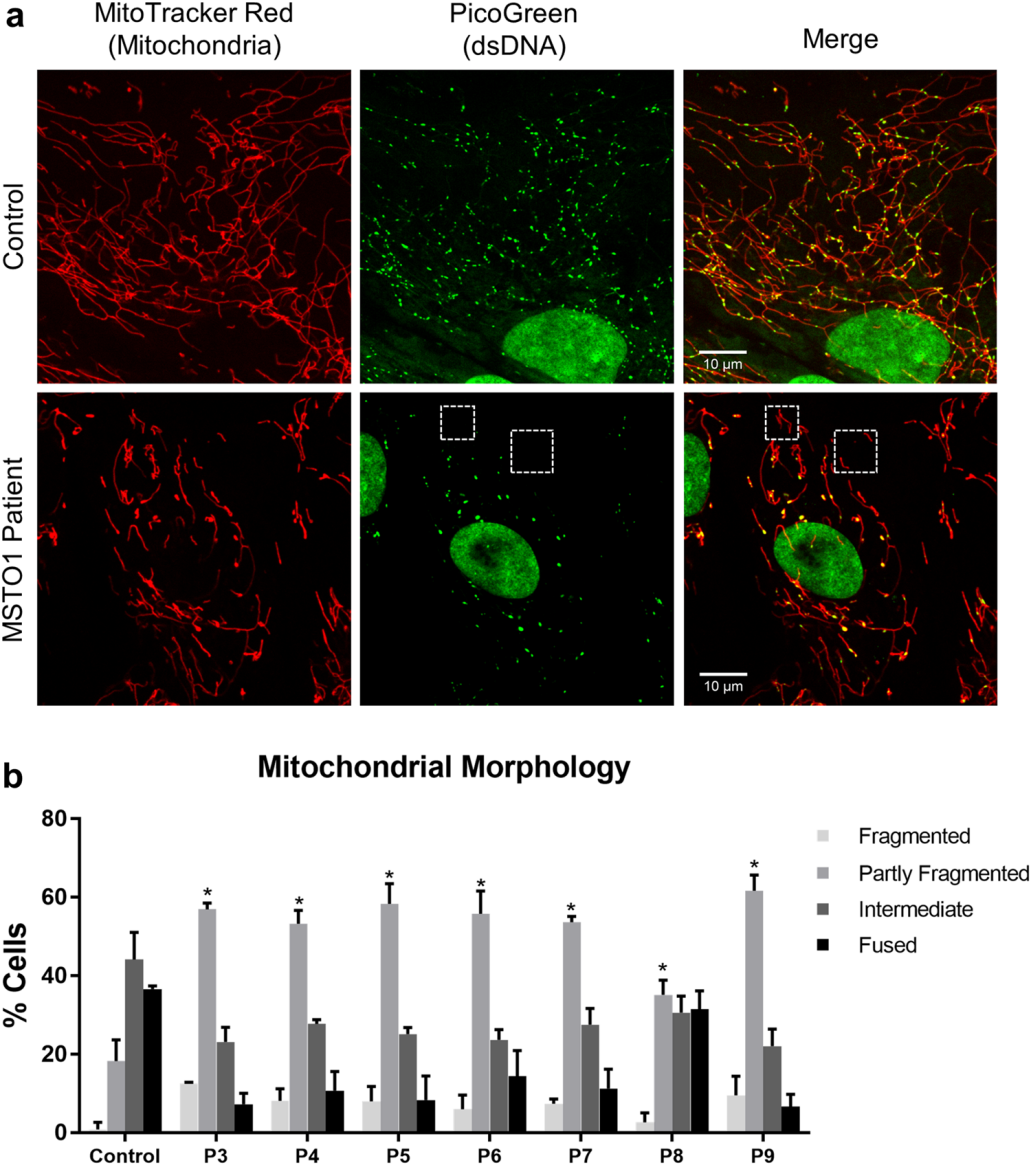
Characteristics of *MSTO1* patient fibroblasts. **a** Representative confocal microscopy images of control and patient cells. Mitochondrial networks in *MSTO1* patient cells are more fragmented and contain fewer but larger mtDNA nucleoids compared to the control cells. Live cells were stained with MitoTracker Red (red, mitochondria) and PicoGreen (green, nuclear and mitochondrial DNA). **b** Quantification of mitochondrial morphology from control and patient cells performed from three independent replicates. Statistical analysis was performed on the number of cells with partly fragmented mitochondrial morphology in control versus patient cells; Student *T* test, *p < 0.05, **p < 0.001

**Fig. 5:**
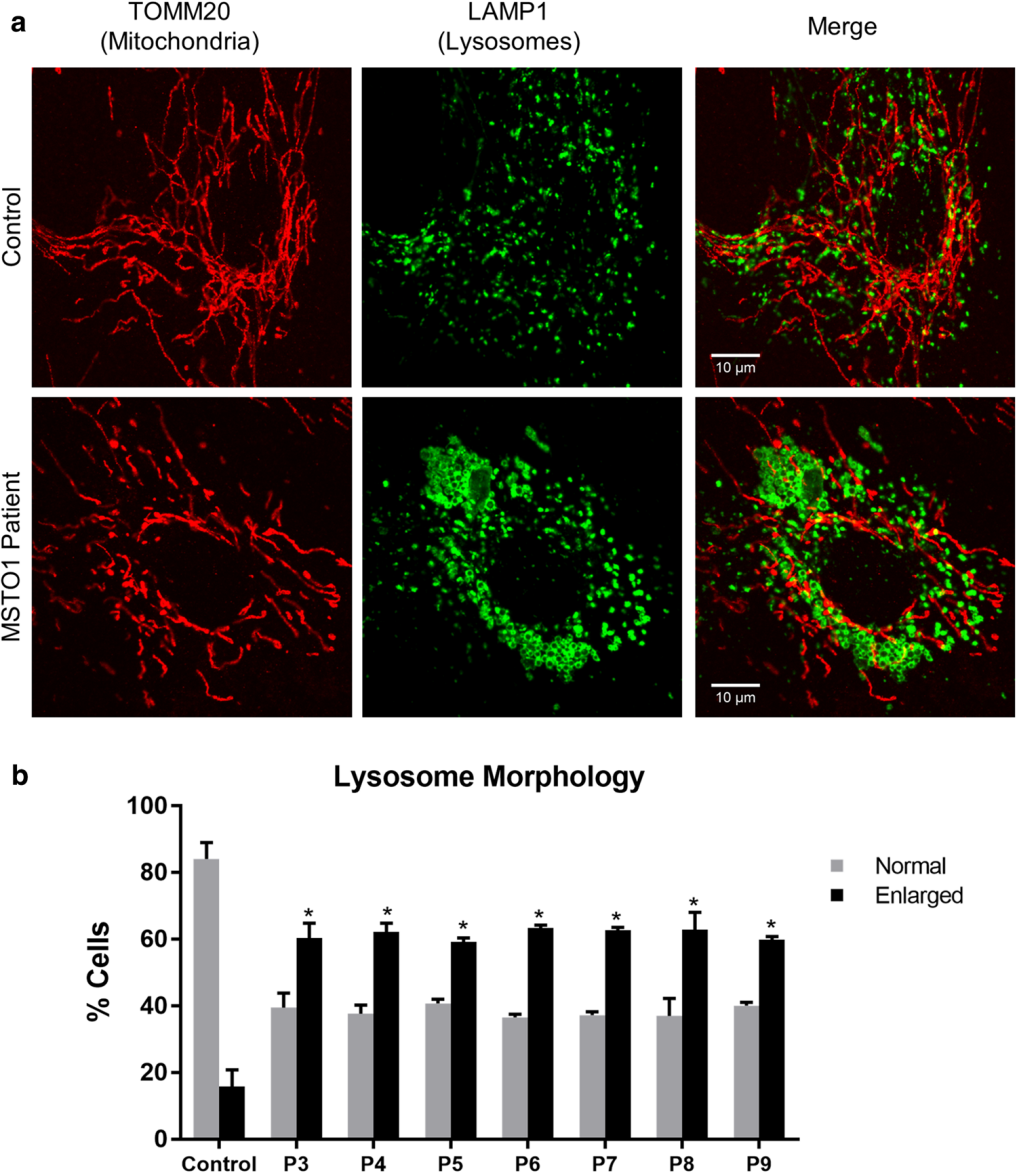
Enlarged lysosomal vacuoles in *MSTO1* patient fibroblasts. **a** Representative confocal images of control and patient cells fixed and stained with antibodies against TOMM20 (red, mitochondria) and LAMP1 (green, lysosomes). Compared to an unaffected control, patient cells contain distinct lysosomal clusters. **b** Quantification of cells containing enlarged lysosomes in control and patient fibroblasts performed from two independent replicates. Statistical analysis was performed; Student *T* test, *p <0.05

Given the established role of MSTO1 as a mitochondrial fusion regulator [16, 36], we examined mitochondrial morphology in seven *MSTO1* patient fibroblasts. Consistent with previous reports, visualized mitochondrial networks in patient fibroblasts were fragmented compared to control fibroblasts (Fig. 4). In addition, we examined lysosomal structures in *MSTO1* patient fibroblast lines by immunofluorescence, as mitochondrial dysfunction can also cause alterations to lysosomes [12]. Compared to unaffected control cells, we observed markedly enlarged lysosomal vacuoles across all *MSTO1* patient lines (Fig. 5), an observation that has not been previously reported in the context of MSTO1 dysfunction [16, 35].

**Fig. 6:**
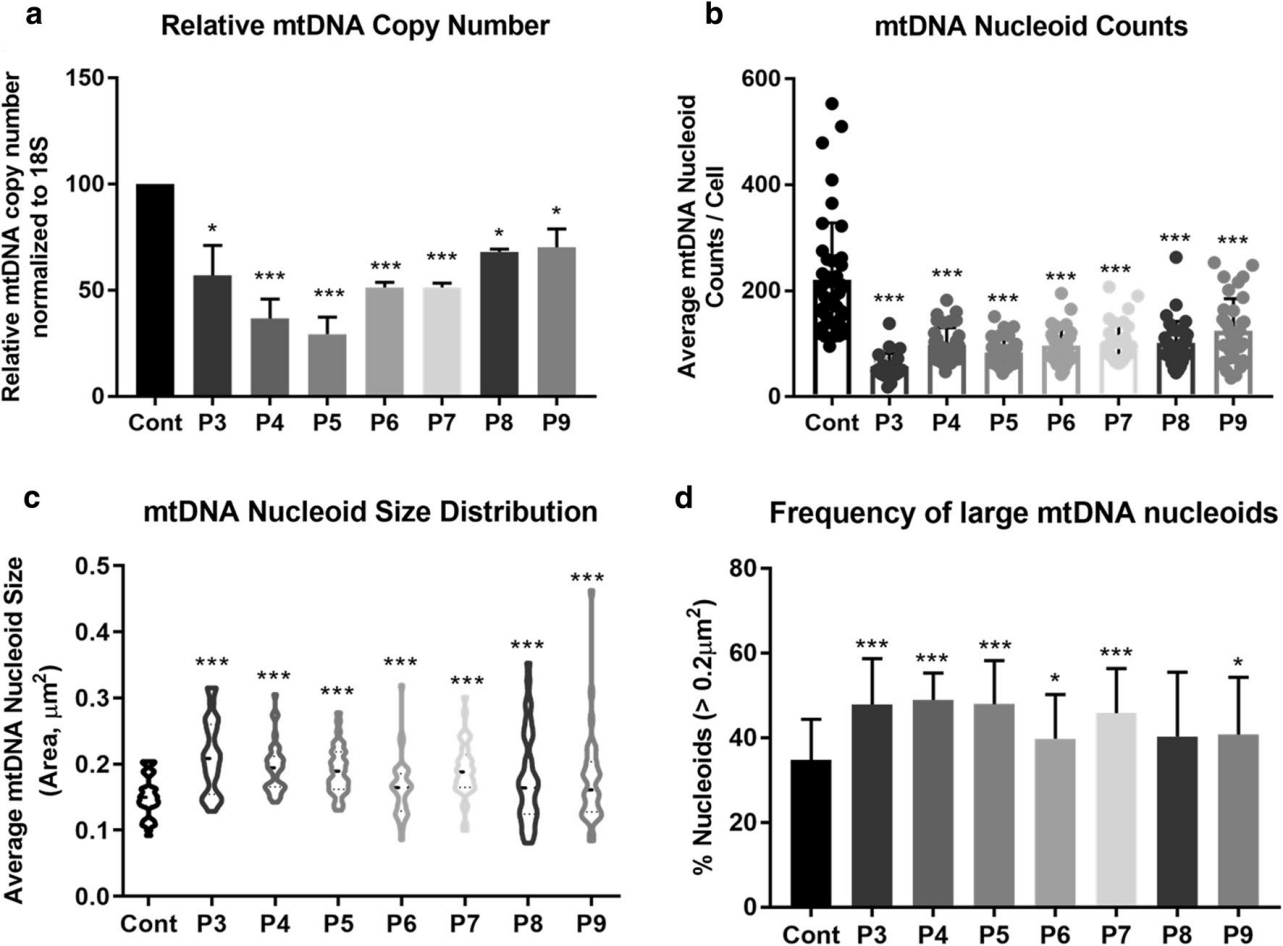
Pathogenic variants in *MSTO1* are linked to mtDNA depletion. **a** Relative mtDNA copy number normalized to the nuclear-encoded 18S gene. Data represent at least three independent biological replicates. **b** Analysis of mtDNA nucleoid counts per cell from 35 cells for each group. **c** Quantification of nucleoid sizes in control and patient cells. Data represent average nucleoid sizes from the same cells as in **b**. Average mtDNA nucleoid size is presented in a violin plot. K–S test was performed to determine statistical significance. **d** Frequency of nucleoids larger than 0.2 µm^2^ in all 35 cells quantified per fibroblast line. Student *T* test was performed as indicated for **a**, **c** and **d**. *p <0.05, **p <0.01, ***p <0.0001

As pathogenic variants in mitochondrial fusion proteins MFN2 and OPA1 have been shown to cause mtDNA depletion [3, 49], we analysed mitochondrial genomes in patient cells. We observed a significant reduction in mtDNA copy number across all fibroblast lines, ranging from 30 to 70% depletion (Fig. 6a). MtDNA nucleoids are nucleoprotein assemblies involved in the organization and segregation of mtDNA. While examining the size and distribution of mtDNA nucleoids within the mitochondrial network in *MSTO1* patient fibroblasts, we found that the patient fibroblasts contained fewer nucleoids, which were larger in size compared to control lines (Fig. 6b, c). Notably, several mitochondrial fragments were devoid of mitochondrial genomes in patient cells (Fig. 4a), a phenotype previously reported in cells lacking fusion regulation [10]. Together, these observations demonstrate significant alterations of the mitochondrial genome in all patient fibroblast lines evaluated. Unfortunately, muscle tissue was not available for further mtDNA content studies.

**Fig. 7:**
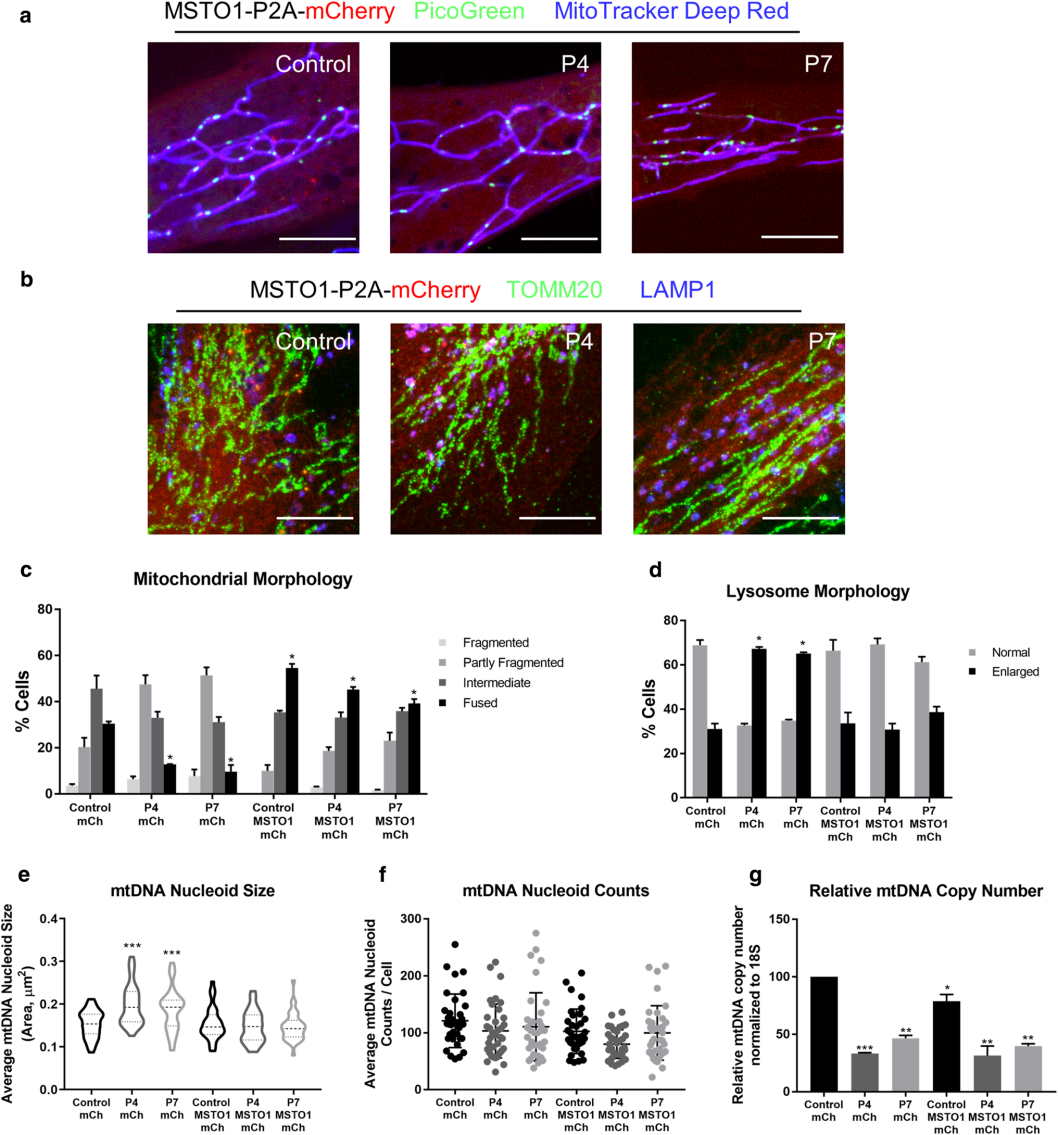
Expression of wild-type MSTO1 rescues cellular phenotypes in *MSTO1* patient fibroblasts. Control, P4 and P7 fibroblast cells were transfected with MSTO1-P2A-mCherry or the mCherry empty vector control. Representative images of fibroblasts transfected with MSTO1-P2A-mCherry, for **a** live cells stained with picogreen and MitoTracker Deep Red, or **b** fixed cells stained with antibodies against TOMM20 (green, mitochondria) and LAMP1 (blue, lysosomes). Scalebars: 10 µm. Transfected fibroblasts, as identified by cytosolic mCherry signal, were characterized as described above for the following cellular phenotypes: **c** mitochondrial morphology, **d** lysosome morphology, **e** average mtDNA nucleoid size, **f** mtDNA nucleoid counts, and **g** relative mtDNA copy number. Student *T* test was performed as indicated for **c**, **d**, **f**, and **g**. K–S test was performed to determine statistical significance for **e**. *p < 0.05, **p < 0.01, ***p < 0.0001

The similarity and consistency of the cellular phenotypes described across all seven *MSTO1* patient fibroblast lines strongly support the notion that loss of MSTO1 function is the underlying cause responsible for these observations. In order to further confirm that the cellular phenotypes were in fact due to the loss of MSTO1, we transiently expressed wild-type MSTO1 in two of the patient cell lines (P4 and P7) (Fig. 7a, b). Similar to previous reports [16, 35], we found that expression of wild-type MSTO1 restored mitochondrial morphology after 48 h (Fig. 7c). Notably, we also observed more fused mitochondrial networks in control cells overexpressing MSTO1, further validating the role of MSTO1 in promoting fusion. In addition, we also see that lysosome abnormalities are restored (Fig. 7d). While we observed a significant rescue in MSTO1 fibroblasts with regard to mtDNA nucleoid size (Fig. 7e), the number of mtDNA nucleoids did not change in MSTO1 fibroblasts (Fig. 7f). A potential confounding factor is that the transfection protocol itself causes a decrease in mtDNA nucleoid counts, which could be masking a rescue. However, mtDNA copy number was also not rescued after only 48 h (Fig. 7g). This incomplete rescue of mtDNA nucleoid abundance and copy number likely reflects the fact that it may take longer than 48 h for the mtDNA copy number to be re-established.

Collectively, our data suggest that various pathogenic variants in *MSTO1* behave in a similar fashion and lead to mitochondrial abnormalities in patient cells, in particular with regard to mtDNA, providing novel mechanistic insight into the disease pathogenesis associated with *MSTO1* mutations.

## Discussion

Bi-allelic pathogenic variants in the nuclear-encoded cytosolic protein *MSTO1* have been reported in 12 patients from 7 families to date [6, 16, 31, 36]. This study characterizes 12 families with an additional 15 affected patients and thus presents the largest single cohort of patients with variants in the cytosolic mitochondrial fusion regulator, *MSTO1*. The series includes several novel pathogenic variants and allows for further delineation of the recessive *MSTO1*-related disease-associated phenotype. We found this phenotype in our cohort to be remarkably consistent with childhood-onset, fairly non-progressive muscle weakness and clinical evidence of corticospinal tract and cerebellar involvement. As a corollary to this clinical presentation, an elevated CK level associated with a histologically dystrophic myopathy and early-onset/congenital yet stable cerebellar atrophy/hypoplasia is seen on testing. Clinical findings are similar to a recently reported small case series of 12 patients with recessive *MSTO1*-related disease [6, 16, 23, 31, 35]. Pigmentary retinopathy was previously observed as part of the recessive phenotype [23, 35]. This finding was not reported in any of the patients in our cohort; however, formal ophthalmologic examination was not pursued in all patients. Arthrogryposis was also previously reported in one patient [23]. While congenital onset hypotonia was noted in three of our patients, no other abnormalities were reported at birth.

The delayed motor development seen in our patients could be due to the cerebellum volume loss, the muscular dystrophy or more likely a combination of both. Review of brain MRI imaging in our cohort demonstrated that the decrease in cerebellar volume is evident in both hemispheres; the volume loss in the vermis is present at a very young age (as observed on the first MRIs obtained) and is non-progressive on follow-up imaging in the majority of patients. Hence, it is possible that the cerebellar volume loss reflects more of a hypoplasia rather than an early-onset progressive atrophy, or a combination thereof. In early cerebellar hypoplasias that interfere with normal cerebellar development, granule cell proliferation deficiency as well as some granular cell loss may underlie an overall smaller cerebellar cortical volume [19]. This may be in keeping with the slight progression of cerebellum volume loss seen in consecutive scans obtained for P12 (p.(Arg345His); p.(Thr324Ile)) at ages 1 and 2 years, which may be disproportionate to the overall brain volume and reflective of a developmental process given that cerebellum growth continues after birth [1]. Without further morphological autopsy data, the relative contributions of hypoplasia versus atrophy will have to remain undetermined. Clinically, it is reassuring that all patients achieved ambulation, which has been maintained at age 52 years in the oldest patient reported to date with *MSTO1*-related disease [P14; p.(Phe217Leu); p.(Arg279Cys)].

One of the most important diagnostic considerations in patients presenting with childhood-onset muscle weakness, elevated CK and structural brain abnormalities with prominent cerebellar involvement, includes the α-dystroglycanopathies (αDGs). The αDGs are a clinical and genetic heterogenous sub-group within the congenital muscular dystrophies (CMDs) that manifest as an early-onset dystrophic muscle disease with central nervous system involvement, including abnormal neuronal migration resulting in cortical malformations as well as impaired synaptic function [17, 37]. Specifically, αDGs caused by mutations in *ISPD* and *GMPPB* may manifest with phenotypes reminiscent of recessive *MSTO1*-related disease [9, 11, 17]. However, hypoglycosylation of α-dystroglycan is a distinctive marker for the αDGs that can be detected using specific antibodies against the matriglycan glycoepitope of α-dystroglycan on muscle immunohistochemistry and western blot, which would be normal in *MSTO1*-related disease. In fact, immunofluorescence analysis of the muscle biopsy from P8 (p.(Phe217Leu); p,(Asp236His) and P9 (p.(Tyr478Cys); missing) showed normal α-dystroglycan glycoepitope staining (data not shown). The other highly relevant differential diagnosis with a reminiscent clinical spectrum is Marinesco-Sjogren syndrome (MSS) caused by bi-allelic mutations in *SIL1*. MSS is characterized by intellectual disability, early onset cataracts, ataxia with cerebellar atrophy and myopathy. The absence of cataracts and severe intellectual disability appear to distinguish *MSTO1*-related disorders from MSS. Approximately 60% of patients with the classic clinical features of MSS harbour *SIL1* pathogenic variants, whereas only 3% (1/37) of those with atypical features have readily identifiable *SIL1* pathogenic variants. Notably, in the study of *SIL1* negative, atypical MSS patients, one patient was ultimately diagnosed with an *AGK*-related mtDNA depletion syndrome [26]. Therefore, it is possible that other atypical MSS patients may also harbour pathogenic *MSTO1* variants. In order to help facilitate an accurate genetic diagnosis, *MSTO1* should be included in targeted next-generation-based neuromuscular, mitochondrial and ataxia-related panels, where *MSTO1* is currently not included.

In this cohort we report six novel *MSTO1* missense variants, a single base pair deletion and a large genomic deletion. Variants are scattered throughout the *MSTO1* gene and do not preferentially impact specific domains. We identified apparent recurring variants in three specific residues (p.(Phe217Leu), p.(Arg279His) and p.(Asp236His/Gly)). The p.(Arg279His) variant was previously reported as pathogenic in three families in heterozygosity with a truncating allele [23, 31]. There is one individual listed in ExAC who is homozygous for the p.(Arg279His) variant, which would be unusual for a childhood-onset disease [29]. It is, therefore, likely that in its own right this may be a much milder pathogenic allele that needs to occur in compound heterozygosity with a more severe allele in order to manifest as early-onset MSTO1-deficiency [23]. As noted, all patients thus presented with a remarkably homogeneous phenotype; therefore, no clear genotype–phenotype correlations emerged for recessive *MSTO1* variants beyond the p.(Arg279His) observation, and the absence of bi-allelic null mutations.

The *MSTO1* gene is part of a large tandem segmental duplication of approximately 240 kb located on chromosome 1q22. This arrangement is the result of an evolutionary duplication event estimated to have occurred 37 million years ago in the human evolutionary lineage [28], which also resulted in the derivation of the pseudogene *MSTO2P***(**NR_024117). This locus generates a long non-coding RNA with a nucleotide identity degree of 99.5% and 98.1% of exonic and intronic regions, respectively [28]. This high sequence similarity results in ambiguity of alignment of the short-read sequences typically generated with next-generation based genetic testing approaches, as the alignment of short reads to their proper genomic location maps equally well with both *MSTO1* and *MSTO2P*. Consequently, the coverage of *MSTO1* is significantly reduced in WES, WGS and RNA-seq data from our patients, resulting in diagnostic challenges. In fact, even with targeted Sanger sequencing it can be challenging to unambiguously sequence *MSTO1* without contribution of the pseudogene sequence. In this context we also report the first presumed multi-exon deletion of *MSTO1*, which, as expected, was not identified through WES. In this patient [P7; (p.(Leu450Phe); deletion)], WES identified an apparently homozygous *MSTO1* missense variant in the absence of consanguinity; however, subsequent WGS and RNA sequencing failed to identify the deletion due to the inherent difficulties of mapping highly homologous regions. Targeted array CGH analysis with exon-level resolution was able to identify the exon 9-14 deletion. Given the duplicated and thus highly similar regions in this analysis, there were only three probes discriminating *MSTO1* from *MSTO2P*; therefore, the mapping of the deletions to *MSTO1* is still ambiguous. Given the diagnostic confidence in the disease phenotype, the reduced MSTO1 protein in this patient’s fibroblasts and the previously identified rare, predicted to be damaging missense *MSTO1* variant, we suspect that the deletion is likely encompassing *MSTO1*. In contrast, in patient P9 (p.(Tyr478Cys); missing), who also presented with the disease phenotype and reduced MSTO1 protein levels, extensive next-generation based-sequencing including array CGH analysis only yielded a single heterozygous rare, predicted to be damaging missense *MSTO1* variant. A pathogenic variant on the other allele was not readily detectable with available technology, suggesting a more complex genomic re-arrangement which may be copy number neutral. Validation work is in progress; however, this work is significantly complicated by the high sequence similarity between the duplicated genomic regions of the locus. Thus proper diagnosis of MSTO1-deficiency may require specialized sequencing strategies, triggered by proper phenotypic recognition through detailed clinical examination, brain MRI and if needed MSTO1 protein analysis in fibroblasts [15].

In all seven novel *MSTO1* fibroblast lines characterized, MSTO1 protein was reduced (Fig. 3a), and mitochondrial network fragmentation was observed (Fig. 4). Consistent with our findings, it has been shown previously that the levels of the mitochondrial fusion proteins MFN1/2 and OPA1 are not affected in *MSTO1* patient cells (Fig. 3b), [16], suggesting that the observed fragmented mitochondrial network phenotype in patient cells is related to MSTO1-deficiency. While defects in mitochondrial fusion have been linked to abnormalities in mtDNA, the specific consequences of pathogenic *MSTO1* variants regarding mtDNA integrity have not been thoroughly investigated thus far [47]. Of the previous MSTO1 studies, three did not investigate mtDNA [16, 31]. Meanwhile, Nasca et al. reported mtDNA depletion in muscle from patient A1 and fibroblasts from patients A1 and A2 (sisters), while mtDNA nucleoid size was not quantified [36]. Analysis of mtDNA copy number across our patient cohort now provides further insights into the role of impaired mitochondrial fusion and mtDNA depletion in *MSTO1*-deficient patient cells. It would be of interest to study this phenomenon in muscle tissue from MSTO1-deficient patients, which unfortunately were not available in this cohort.

While the mechanistic link between mitochondrial fusion/fission dynamics and loss of mtDNA content remains unresolved, the present study adds another mitochondrial fusion protein, MSTO1 to the list of mitochondrial dynamics proteins that are implicated in the maintenance of mtDNA content. Our observation of enlarged nucleoids using PicoGreen staining is of interest in this context. The enlarged nucleoids could reflect changes in the topology of mtDNA that affect the ability of the dye to intercalate [20]. Alternatively, impaired segregation of mtDNA nucleoids, which typically contain only a single copy of the mtDNA genome [27], could lead to multiple mtDNA molecules within close spatial proximity that appear as larger nucleoids [8]. We favour the latter hypothesis given that loss of fusion is known to impair nucleoid distribution with some mitochondrial fragments containing a relatively higher concentration of mtDNA molecules while others completely lack mtDNA [10], which we also observe in *MSTO1* fibroblasts (Fig. 4a). Meanwhile, recent work suggesting that reduced fusion causes mtDNA depletion due to insufficient distribution of the mtDNA replication machinery [44] is also consistent with our findings in *MSTO1* fibroblasts. In particular, the fact that we see a full recovery of mitochondrial and lysosome morphology at 48 h, but only a partial recovery of mtDNA (in the form of restoration of nucleoid size), further suggests that impairments in mitochondrial dynamics are upstream of mtDNA depletion in *MSTO1* patient fibroblasts. Nonetheless, the exact link between mtDNA copy number and enlarged nucleoids remains unresolved.

It is also notable that mitochondrial fission was recently shown to be important for mtDNA nucleoid segregation following mtDNA replication [30] and that impairments in fission are known to lead to enlarged nucleoid structures [8]. Taken together, these observations provide further evidence that mitochondrial fusion and fission are important for mtDNA segregation and distribution. We posit that altered distribution and segregation of mtDNA (*i.e.* nucleoid clumping), in conjunction with reduced mitochondrial fusion, impairs mtDNA maintenance leading to mtDNA depletion.

Importantly, the degree of mtDNA depletion we see in *MSTO1* patient fibroblasts (30–70% of the normal content) is consistent with the levels of mtDNA depletion reported in fibroblasts from OPA1 or MFN2 patients [41, 46, 49] as well as in mtDNA depletion syndromes (Fig. 6) [38]. The clinical significance of this observation requires further studies as the remarkably consistent and homogenous phenotype of recessive *MSTO1*-related disease reported within our cohort is in contrast to the frequently highly variable clinical spectrum observed in mitochondrial diseases with childhood onset due to either nuclear DNA or mtDNA pathogenic variants. Thus, the consequences of abnormal mitochondrial dynamics caused by recessive *MSTO1* pathogenic variants may be less susceptible to acutely variable changes in metabolic demands, while likely incorporating a developmental component given the early clinical manifestations and neuroimaging abnormalities. This consistent phenotype of MSTO1-deficiency reported to date also seems in contrast to the single family with a dominantly acting *MSTO1* variant in whom psychiatric manifestations including schizophrenia and autism were leading clinical features, while muscle weakness was mild and cerebellar involvement was not reported [16], thus suggesting a different underlying pathogenic mechanism. It is notable that the pathogenic recessive variants in *MSTO1* investigated to date seem to impair protein stability in fibroblasts, which is consistent with a loss of function mechanism, while not providing further insight into functional subdomains of the protein. Since haploinsufficiency, as is present in some of the heterozygous parents in our cohort, does not cause a clinical phenotype, the mechanism for the dominantly acting heterozygous mutation may be dominant-negative in nature with perhaps different physiological consequences [16].

Despite evidence for the role of MSTO1 in mitochondrial fusion, the molecular structure of the MSTO1 protein and the exact mechanism by which MSTO1 performs this function directly or via mediators are still unknown. Our findings that bi-allelic loss-of-function variants in *MSTO1* result in fragmented mitochondrial networks with mtDNA depletion and nucleoid abnormalities highlight a previously unappreciated role for MSTO1 in the maintenance of the mitochondrial genome. Taken together, our findings newly characterize MSTO1-deficiency as a syndrome of both impaired mitochondrial fusion as well as of mtDNA depletion, while clinically manifesting with a remarkably consistent phenotype.

## Electronic supplementary material

Below is the link to the electronic supplementary material.

Supplementary material 1 (PDF 153 kb)
