# Supplementary material for: MSTO1 mutations cause mtDNA depletion, manifesting as muscular dystrophy with cerebellar involvement
Source: Acta Neuropathol. 2019 Aug 29;138(6):1013–31. doi: 10.1007/s00401-019-02059-z (PMC6851037; doi:10.1007/s00401-019-02059-z)
Supplement: Supplementary file 1 — Supplementary material 1 (PDF 153 kb) [file 401_2019_2059_MOESM1_ESM.pdf]

Supplementary methods

Whole exome sequencing details

WES for F1 was pursued on P1-P3 and an unaffected brother as previously described (Bernier, Caluseriu et al. 2012).

WES for F2 (P4 and P5 and unaffected parents), was pursued by capturing the exonic regions and flanking splice junctions of the genome using the IDT xGen Exome Research Panel v1.0. Massively parallel (NextGen) sequencing was done on an Illumina system with 100bp or greater paired-end reads. Reads were aligned to human genome build GRCh37/UCSC hg19, and analyzed for sequence variants using a custom-developed analysis tool. Additional sequencing technology and variant interpretation protocol has been previously described (Retterer, Juusola et al. 2016).

Trio whole exome sequencing (WES) for P6-P10 was performed at the Broad Institute (Boston, USA) using Standard Germline Exome version 5, handled by the Genomics Platform's Core Exome/RNA product team. The process includes sample preparation (Illumina Nextera), hybrid capture (Illumina Rapid Capture Enrichment 37Mb target), sequencing (Illumina, 150bp paired reads) and identification quality control check. The hybrid selection libraries typically meet or exceed 85% of targets at 20x, comparable to ~55x mean coverage.

Singleton WES for P11 was performed at the Broad Institute (Boston, USA) using Standard Germline Exome which includes sample plating, library preparation, hybrid capture, sequencing (76bp paired reads), sample identification quality control check, and data storage. This product utilizes the Agilent Sure-Select Human All Exon v2.0, 44Mb baited target with the Broad in-

solution hybrid selection process. With >5Gb PF of data our hybrid selection libraries typically meet or exceed 80% of targets at 20x (+/- 5%) and a mean target coverage of >80x (+/- 5%).

Singleton WES for P12 was pursued through an outside commercial laboratory.

Exome sequencing for P13 & P14 was performed as part of the MyoSeq Project as previously reported (Johnson, Töpf et al. 2017).

Trio WES for P15 was pursued through an outside commercial laboratory. Library preparation was performed utilizing the KAPA Biosystems kit (KAPA Biosystems, Woburn, MA.) Samples were enriched using IDT xGen exome panel and sequenced to a minimum of 7 Gb of 2x125 paired end reads for a mean of 80x average coverage or greater on the Illumina HiSeq 4000. Bidirectional sequence is assembled, aligned to reference gene sequences based on human genome build GRCh37/UCSC hg19, and analyzed using custom-developed software, RUNES and VIKING (www.pediatricgenomicmedicine.com).

References

Bernier, F. P., O. Caluseriu, S. Ng, J. Schwartzentruber, K. J. Buckingham, A. M. Innes, E. W. Jabs, J. W. Innis, J. L. Schuette, J. L. Gorski, P. H. Byers, G. Andelfinger, V. Siu, J. Lauzon, B. A. Fernandez, M. McMillin, R. H. Scott, H. Racher, F. C. Consortium, J. Majewski, D. A. Nickerson, J. Shendure, M. J. Bamshad and J. S. Parboosingh (2012). "Haploinsufficiency of SF3B4, a component of the pre-mRNA spliceosomal complex, causes Nager syndrome." Am J Hum Genet **90**(5): 925-933.

Johnson, K., A. Töpf, M. Bertoli, L. Phillips, K. G. Claeys, V. R. Stojanovic, S. Perić, A. Hahn, P. Maddison, E. Akay, A. E. Bastian, A. Łusakowska, A. Kostera-Pruszczyk, M. Lek, L. Xu, D. G. MacArthur and V. Straub (2017). "Identification of GAA variants through whole exome sequencing targeted to a cohort of 606 patients with unexplained limb-girdle muscle weakness." Orphanet Journal of Rare Diseases **12**(1): 173.

Retterer, K., J. Juusola, M. T. Cho, P. Vitazka, F. Millan, F. Gibellini, A. Vertino-Bell, N. Smaoui, J. Neidich, K. G. Monaghan, D. McKnight, R. Bai, S. Suchy, B. Friedman, J. Tahiliani, D. Pineda-Alvarez, G. Richard, T. Brandt, E. Haverfield, W. K. Chung and S. Bale (2016). "Clinical application of whole-exome sequencing across clinical indications." Genet Med **18**(7): 696-704.
